# Temozolomide 3 weeks on and 1 week off as first-line therapy for recurrent glioblastoma: phase II study from gruppo italiano cooperativo di neuro-oncologia (GICNO)

**DOI:** 10.1038/sj.bjc.6603376

**Published:** 2006-10-03

**Authors:** A A Brandes, A Tosoni, G Cavallo, R Bertorelle, V Gioia, E Franceschi, M Biscuola, V Blatt, L Crinò, M Ermani

**Affiliations:** 1 Department of Medical Oncology, Bellaria Hospital, 40139 Bologna, Italy; 2 Department of Medical Oncology, Istituto Oncologico Veneto-IRCCS, 35128 Padova, Italy; 3 Servizio di Immunologia e Diagnostica Molecolare Oncologica – Istituto Oncologico Veneto-IRCCS, 35128 Padova, Italy; 4 Dipartimento Farmaceutico – Azienda USL of Bologna, 40139 Bologna, Italy; 5 Department of Medical Oncology Perugia Hospital, 06100 Perugia; 6 Department of Neurological Sciences, Azienda Ospedale-Università of Padova, 35100 Padova, Italy

**Keywords:** clinical trials, glioblastoma, *MGMT*, prolonged temozolomide

## Abstract

The efficacy of temozolomide strongly depends on O^6^-alkylguanine DNA-alkyl transferase (AGAT), which repairs DNA damage caused by the drug itself. Low-dose protracted temozolomide administration can decrease AGAT activity. The main end point of the present study was therefore to test progression-free survival at 6 months (PFS-6) in glioblastoma patients following a prolonged temozolomide schedule. Chemonaïve glioblastoma patients with disease recurrence or progression after surgery and standard radiotherapy were considered eligible. Chemotherapy cycles consisted of temozolomide 75 mg/m^2^/daily for 21 days every 28 days until disease progression. O^6^-methyl-guanine-DNA-methyl-tranferase (*MGMT*) was determined in 22 patients (66.7%). A total of 33 patients (median age 57 years, range 31–71) with a median KPS of 90 (range 60–100) were accrued. The overall response rate was 9%, and PFS-6 30.3% (95% CI:18–51%). No correlation was found between the *MGMT* promoter methylation status of the tumours and the overall response rate, time to progression and survival. In 153 treatment cycles delivered, the most common grade 3/4 event was lymphopoenia. The prolonged temozolomide schedule considered in the present study is followed by a high PFS-6 rate; toxicity is acceptable. Further randomised trials should therefore be conducted to confirm the efficacy of this regimen.

Glioblastoma multiforme (GBM), the most aggressive brain tumour in adults despite, advances in diagnosis and treatment made over the past two decades, is still incurable (Brandes, 2003). Every effort should, therefore, be made to develop new approaches that maximise an active drug, such as temozolomide (TMZ), which is the backbone in the treatment of brain tumours. However, the efficacy of this therapy is limited by the presence of intrinsic or acquired resistance mechanisms. Temozolomide exerts its activity by means of DNA methylation at the N^7^ and O^6^ position of guanine and at the O^3^ position of adenine (Denny *et al*, 1994). Although O^6^-methylguanine accounts for few adducts formed by TMZ, it plays a critical role in the cytotoxicity of the drug. The DNA repair protein, O^6^-alkyl-guanine-DNA-alkyltransferase (AGAT), encoded by the *MGMT* gene, reverts resistance to TMZ by removing cytotoxic methyl adducts from the O^6^ position of guanine. As AGAT removes methyl adducts from DNA via a suicide mechanism, it has also been suggested that protracted exposure to an alkylating agent may not only saturate AGAT copies available within cancer cells, but may also inactivate the new molecules while they are being synthesised, thus leading to AGAT ‘depletion’ and overcoming the inherent resistance of glioma cells.

Tolcher *et al* (2003) showed that mean AGAT activity decreased by 63% in peripheral blood mononucleal cells (PBMC) after 14 days of protracted TMZ treatment, and by 73% after 21 days; these low levels persisted up to day 28. In the present study, AGAT activity in PBMC has been used as a pharmacodynamic surrogate end point for AGAT depletion, although changes in AGAT activity in PBMC may not reflect changes in tumour tissues. A phase I study was performed using this TMZ schedule (3 weeks on/1 week off) in patients with advanced-stage solid malignancies (Denis *et al*, 2000). Dose-limiting toxicity was thrombocytopenia, the maximum-tolerated dose being 100 mg/m^2^. However, as toxicity was detected only after two courses of treatment, no definitive conclusions could be drawn regarding the long-term toxicity of the regimen at this dosage. The aim of the present phase II study was therefore to evaluate the effect of a prolonged TMZ schedule of 75 mg/m^2^/daily for 21 days every 28 days in relation to progression-free survival at 6 months (PFS-6), response, toxicity and any correlation with *MGMT* gene promoter methylation status, in patients with recurrent or progressive GBM.

## MATERIALS AND METHODS

### Eligibility

Criteria for eligibility were: histological diagnosis of GBM, age ⩾18 years, Karnofsky Performance Score (KPS) ⩾60, normal baseline counts for neutrophils ⩾1500/*μ*l and platelets ⩾100 000/*μ*l; transaminases and alkaline phosphatase levels ⩽1.5 times the upper normal limits; bilirubin and creatinine levels ⩽1.25 times the upper normal limits; previous surgery followed by standard radiotherapy (60 Gy/30 fractions). Unequivocal evidence of disease recurrence or progression at gadolinium-enhanced MRI neuroimaging was also required. Patients accrued had at least one enhancing measurable lesion with a diameter of ⩾2 cm, evaluated at least 3 months after the end of radiotherapy. Brain imaging, performed within 2 days after surgery, showing residual disease with the above characteristics was mandatory for patients undergoing repeat surgery for recurrence. All patients accrued had been on a stable dose of corticosteroids for at least 2 weeks before initiation of therapy. Patients with childbearing potential were to use effective contraception. Pregnant or breast-feeding patients were ineligible, as were patients who had previously received cytotoxic therapy, presented active infection or other uncontrolled diseases, psychiatric disturbances and/or a history of cancer other than resected nonmelanoma skin cancer or carcinoma *in situ* of the uterine cervix.

Availability of tumour specimens to perform the assessment of *MGMT* promoter methylation status was required for all patients. The study, approved by the Institutional Review Boards of all participating centres, was conducted according to the principles of the Declaration of Helsinki and the rules of Good Clinical Practice. All patients signed a form giving their fully informed consent to participate.

### Treatment regimen

All patients were given TMZ 75 mg/m^2^/daily for 21 days every 28 days. No dose escalation was allowed. Patients fasted for at least 2 h before, and 2 h after, TMZ administration. As continuous daily TMZ can cause lymphopoenia, potentially increasing the risk of opportunistic infections, patients received oral trimethoprim-sulphamethoxazole to prevent *Pneumocystis carinii* pneumonia if the lymphocyte count fell to <500/*μ*l (Kovacs and Masur, 2000). Antiemetic prophylaxis with metoclopramide or a 5-hydroxytriptamine3 antagonist was also given.

### Dose modifications

Patients were closely monitored for toxicity throughout cycles, all adverse events being recorded and graded according to the common toxicity criteria of the National Cancer Institute, version 3.0. (http://ctep.cancer.gov/forms/C TCAEv3.pdf).

Haematology was performed weekly, while complete biochemistry was assessed once per cycle, preferably on day 28. Chemotherapy was given if neutrophils were ⩾1500/*μ*l, lymphocytes ⩾500/*μ*l and platelets ⩾100 000/*μ*l; otherwise treatment was delayed for a maximum of 3 weeks until adequate recovery. If blood counts analysed throughout 3 weeks were still unsatisfactory, treatment was stopped. In cases of ⩾G3 haematological toxicity at nadir or reversible G3 nonhaematological toxicity (except for nausea/vomiting), TMZ was reduced by 25%. If G4 haematological or G3 nonhaematological toxicity reappeared notwithstanding dose reductions, or if any type of nonhaematological G4 toxicity was observed, chemotherapy was interrupted.

The use of growth factors in order to maintain high blood counts and to administer chemotherapy at fixed intervals was proscribed. Patients were kept at the lowest corticosteroid dosage allowed in relation to their neurological status.

### Efficacy measures

Progression-free survival was evaluated from the start of chemotherapy to progression; median survival (MST) was calculated from the start of chemotherapy to death for any reason. In this intent-to-treat study, data on all registered patients were included in the statistical analysis.

PFS, PFS-6 and MST were calculated using the Kaplan–Meier method (Kaplan & Meier, 1958); differences in progression and overall survival (OS) were evaluated by the log-rank test for statistical significance.

Patients were evaluated for response using clinical and neurological examinations (performed monthly before each cycle) and MRI or CT neuroimaging performed every two cycles, or earlier if indicated, according to Macdonald's criteria (Macdonald *et al*, 1990). Neurological status was assessed by considering signs and symptoms possibly correlated with progression, as compared to the previous examination; each variation in daily corticosteroids dosage was recorded.

Responses were confirmed as complete or partial if they were constant at subsequent scans obtained at least 4 weeks apart from each other. An independent central review of CT and MRI scans was made for patients achieving complete (CR) or partial (PR) response or stable disease (SD), evaluated by local investigators. Patients were withdrawn if they had progressive disease, unacceptable toxicity, or retracted their consent.

### DNA extraction and methylation-specific polymerase chain reaction

DNA from 10 mm paraffin sections of cerebral lesion was modified by sodium bisulphite, which converts unmethylated cytosine to uracil, according to the procedure of Herman *et al* (1996). Modified DNA was submitted for methylation specific PCR (MSP) by a nested-PCR protocol (Palmisano *et al*, 2000). As the quality of DNA obtained from formalin-fixed paraffin-embedded tumour tissue affects the success rate of MSP, in some cases *MGMT* methylation status was determined by a different nested-MSP approach, with a first pair of primers to obtain smaller amplicons (129 bp), for which forward and reverse primers have been described (Palmisano *et al*, 2000; van Engeland *et al*, 2003).

### Statistical analysis

The trial was a phase II study, with PFS-6 percentage as the main end point. According to the Minimax design (Simon, 1989), our study, with its sample size of *n*=33, had a 5% probability of rejecting (*α*) the hypothesis of a PFS-6 10% (P_0_) and a 90% probability of accepting (1-*β*) the hypothesis of a PFS-6 of 30% (P_1_). If two, or fewer of the first 22 patients were progression free at 6 months, PFS-6 would be considered <10% and the study terminated. Otherwise, the study would be completed, the accrual target being 33 patients. If six or fewer of the 33 patients were progression-free at 6 months, then no further investigation of the treatment regimen was considered warranted. PFS, PFS-6 and OS were calculated using the Kaplan–Meier method (Kaplan and Meier, 1958), and differences in progression and survival in relation to prognostic factors were evaluated with the log-rank test. Student's *T* and the Mann–Whitney *U*-tests were used to analyse normally and non-normally distributed variables between groups. All calculations were performed using S-PLUS software (MathSoft Inc., Seattle, WA, USA).

## RESULTS

### Patient characteristics

From November 2003 to September 2005, 33 patients (13 females; median age 57, range 31–71 years; median KPS 90, range 60–100) were enrolled; their characteristics are reported in Table 1. All patients were evaluated for drug activity and toxicity.

### Methylation specific PCR analysis

Methylation specific PCR (MSP) analysis was performed in all 33 patients enrolled in the trial. However, results were evaluable only in 22 patients, with an MSP success rate of 66%; this finding is comparable to those reported by other authors (Hegi *et al*, 2005).

Among 22 patients for whom MSP was evaluable, 10 (45.5%) presented *MGMT* promoter methylated and 12 unmethylated (54.5%) status. No differences were found between patients with *MGMT* promoter methylated and those with unmethylated status for age (*P*=0.16), gender (*P*=0.39), performance status (PS) (*P*=0.72), time-intervals between surgery and start of chemotherapy (*P*=0.21), or between the end of radiotherapy and start of chemotherapy (*P*=0.15). All patients had undergone one surgical procedure and full-dose radiotherapy, completed at least 3 months previously, but none had been given cytotoxic or cytostatic drugs.

### Progression-free survival

All patients were followed-up to disease progression. The percentage of patients without progression at 6 months (PFS-6) was 30.3% (95%CI: 18–51%). Overall, the median PFS was 16.1 weeks (95%CI: 12.4–27.3), 15.6 weeks (CI: 11–NA) and 11.9 weeks (CI: 10.3–31.6) in patients with *MGMT* promoter methylated status and in patients with unmethylated *MGMT* promoter status, respectively. No significant differences were found between PFS, evaluated using the log-rank test, in relation to age (*P*=0.42), KPS (*P*=0.77), type of surgery (*P*=0.58) and *MGMT* promoter methylated or unmethylated status (*P*=0.86).

### Response

Among the 33 evaluable patients, one CR (3%) and two PR (6%) were obtained, with an overall response rate of 9% (CI: 0–18.8%). Seventeen patients had SD (51%; CI: 34.4–68.6%). All radiological responses were confirmed by an independent centralised review, and stable or decreased steroid dosage was confirmed in all patients on recording responses, which had a median duration of 30.4 weeks, the median duration of disease stabilisation being 25.6 weeks. No correlations were found between response to therapy and age (*P*=0.32), KPS (*P*=0.96), time-interval between surgery (*P*=0.19) or end of radiotherapy (*P*=0.38), *MGMT* promoter methylated or unmethylated status (*P*=0.63) in responders (CR+PR) and SD patients *vs* PD patients.

### Overall survival

**Figure 1:**
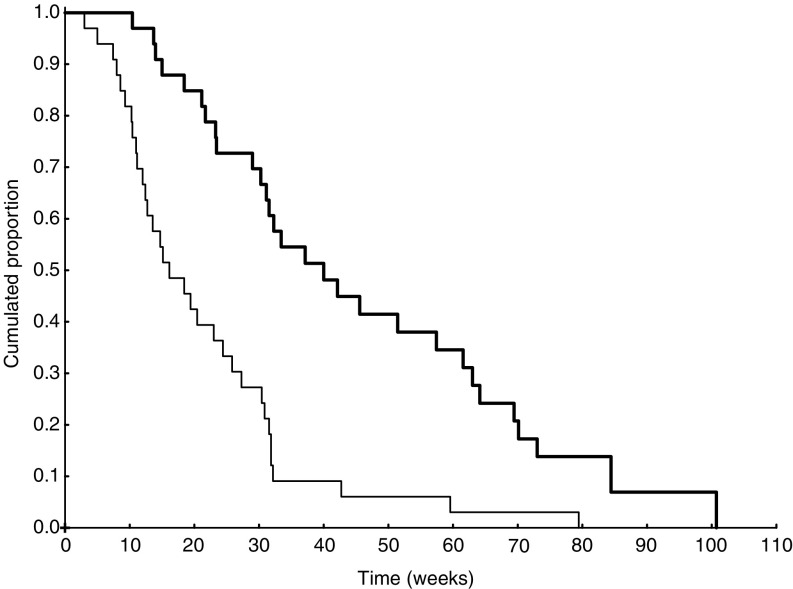
Thin line PFS. Thick line: OS.

Median survival as from the start of chemotherapy was 40 weeks (CI: 31–63), although this outcome may have been influenced by second-line treatments, 12 patients being treated with nitrosourea-based regimens, 3 with carboplatin and etoposide, 3 with new experimental drugs, and 15 with no other treatments. In all, 73% (CI: 59–90%) and 38% (CI: 24–59%) of the patients were alive at 6 and 12 months, respectively. Median survival and percentage of patients alive at 1 year in cases with methylated *MGMT* promoter status were 48.2 weeks (CI: 29–NA) and 50% (95% CI: 27–93%), respectively; in patients with unmethylated *MGMT* promoter status, these figures were 34.7 weeks (95% CI: 30–64.1) and 21.4% (CI: 8–58%), respectively. Only KPS (<90 *vs* ⩾90) was related to survival (*P*=0.05) (Figure 1).

### Toxicity

A total of 153 treatment cycles were given to 33 patients, the median number of cycles per patient was three (range 1–15). The most common adverse event was lymphopoenia (Table 2). As the cycles were delivered, a clear trend toward an increase in lymphopoenia was observed, being present in 30% of patients during the first three cycles, and in 55% of the patients in the subsequent cycles.

Although none of the patients had pneumocistis carinii pneumonia, five (13.6%) had grade 1–3 infections (one herpes zoster, one urinary infection and three upper respiratory tract infection) associated with lymphopoenia grade 1 (one patient), grade 2 (two patients) and grade 3 (two patients). The most commonly found nonhaematological symptoms and signs of toxicity were constipation and grade 1–3 asymptomatic transaminase increase, observed in seven patients (21.2%). Eight cycles (5.2%) were delayed for a median time of 1 week (recovery after 1–7 weeks) due to grade 3 lymphopoenia (25%), grade 2 infections (25%) and grade 4 neutropenia (25%).

The most common reason for drug discontinuation was disease progression, which occurred in 93.9% of patients. One patient died of pulmonary embolism, probably unrelated to TMZ administration; one discontinued treatment due to prolonged (7 weeks) grade 4 pancytopenia.

Based on the supposition that *MGMT*, a potent drug resistance gene, might be implicated in the protection of haematopoietic stem cells during chemotherapy (Gerson, 2004) a search was made for a correlation between *MGMT* promoter status and toxicity. However, *MGMT* promoter methylation status was determined on tumour samples, and not in blood, thus indicating only an indirect correlation between *MGMT* and toxicity. Grade 3–4 leukopenia was observed in three patients with methylated *MGMT* promoter and in one patient with unmethylated *MGMT* promoter, lymphopoenia grade 3 was observed in four patients with methylated *MGMT* promoter, and in one with unmethylated *MGMT* promoter.

## DISCUSSION

In GBM patients with first recurrence, TMZ is active at a standard single daily dose of 150–200 mg/m^2^, administered for five days every 4 weeks. In the three available studies investigating this regimen in this setting, PFS-6 was 21% (Yung *et al*, 2000) (CI:13–29%), 18% (Brada *et al*, 2001) (CI:11–24%), and 24% (Brandes *et al*, 2002) (CI:14–42%). As the antitumour activity of TMZ depends on the level of AGAT within tumour cells, several trials have aimed to deplete AGAT via a continuous dosing schedule (Tolcher *et al*, 2003). In their phase II study with continuous TMZ administration at a dosage of 75 mg/m^2^/day over a 6-week period with 4 weeks' rest, in 28 pretreated GBM patients, Khan *et al* achieved a PFS-6 of 19%, a median PFS 2.3 months and zero responses. This schedule thus allowed a higher dose intensity (1260 mg/m^2^) over 4 weeks than the standard schedule (1000 mg/m^2^) with good tolerability, although the results did not support its use in patients with recurrent GBM. Wick *et al* (2004) treated 21 GBM patients with a different 28-day period continuous schedule (TMZ at 150 mg/m^2^ delivered for 1-week on/1-week off) with a drug intensity of 2100 mg/m^2^ and AGAT (Tolcher *et al*, 2003) was depleted: a PFS-6 of 48% was obtained (43% patients were pretreated with 1–2 chemotherapy regimens) with modest toxicity. However, on using the same regimen, Chinot *et al* (2005) obtained a PFS-6 of only 21% in 29 inoperable previously untreated GBM patients.

We treated recurrent chemonaïve GBM patients with a continuous TMZ schedule for 21 days every 28 days, and a PFS-6 of 30.3% was obtained. Our findings may have been influenced by several factors: chemotherapy administered at relatively low doses while following a frequent metronomic schedule may have optimised the antiangiogenic effect of cytotoxic agents, providing better results than an intensified weekly schedule (Kurzen *et al*, 2003). Moreover, a high-dose intensity (1575 mg/m^2^) was achieved and AGAT depletion was more prolonged than in a weekly schedule, in which recovery of AGAT activity begins immediately after 7 days (Tolcher *et al*, 2003).

The results reported in studies on *MGMT* gene promoter status are controversial. It has been maintained that *MGMT* promoter methylation assessed by MSP is associated with a longer survival in GBM patients treated with radiotherapy and TMZ (18.2 *vs* 12.2 months, *P*<0.001) (Hegi *et al*, 2005). However, Paz *et al* (2004) reported that *MGMT* promoter methylation was correlated with response to standard TMZ, but not with OS. Findings made using alternative methods (immunohistochemistry or activity tests) are contradictory, some confirming (Friedman *et al*, 1998) and others denying (Middleton *et al*, 1998) a relationship with time to progression or response to TMZ.

To our knowledge, ours is the first trial to study correlations between *MGMT* promoter methylation status, assessed by MSP, and treatment outcome with a continuous TMZ schedule. Findings for/MSP were available in 22 (66.7%) of the 33 patients enrolled in the study; this percentage is similar to those reported (59–67%) in MSP studies made by other authors (Hegi *et al*, 2004; Hegi *et al*, 2005). In our study, an overall median PFS of about 4 months and a PFS-6 of 30.3% were obtained: these results appear superior to those obtained with the standard TMZ schedule. In 22 patients for whom *MGMT* promoter status was available, no significant difference was found between *MGMT* promoter methylated or unmethylated patients for median PFS and PFS-6 (15.6 weeks and 20% *vs* 11.9 weeks and 21.4%). This suggests that AGAT depletion achieved with protracted TMZ increases the sensitivity of unmethylated tumours to TMZ, leading to an increase in PFS and OS which would have been less than that obtained by us if these patients had been treated with the traditional TMZ schedule. However, this observation, based on a small number of patients, should be demonstrated by findings from prospective studies with patients stratified according to methylation status, as in the ongoing phase III RTOG/EORTC study.

The standard TMZ schedule (Brada *et al*, 2001; Yung *et al*, 2000) incurs higher grade 3/4 thrombocytopenia (7–10% *vs* 3) and lower grade 3/4 neutropenia (4–4.5% *vs* 12) rates than our 3-week on/1-week off schedule. Cumulative lymphopoenia was observed in 45.4% of our patients, this outcome being comparable to those reported by other authors following other protracted schedules (Brock *et al*, 1998; Khan *et al*, 2002; Su *et al*, 2004; Wick and Weller, 2005). This high incidence of lymphopoenia may have been due in part to the concomitant administration of steroids: 75% of patients received both steroids and continuous TMZ. The standard TMZ schedule rarely leads to lymphopoenia, no such cases being reported in 250 GBM patients treated in phase II–III trials (Yung *et al*, 2000; Brada *et al*, 2001).

Although the number of severe haematologic events observed by us may be of limited statistical power, we observed an association between grade 3 lymphopoenia, grade 4 haematological toxicity and *MGMT* status. Based on experimental evidence indicating low AGAT activity in bone marrow CD34 cells (Gerson *et al*, 1996), it may be suggested that haematological progenitors are especially sensitive to strategies that inactive AGAT: when O^6^ benzylguanine, an AGAT specific inhibitor, is added to BCNU, haematological toxicity increases (Friedman *et al*, 2000). A correlation between AGAT activity in PBMC and haematological toxicity has been demonstrated by Tolcher *et al* (2003) in patients treated with a prolonged schedule. However, in our study *MGMT* promoter methylation status was assessed only on tumour samples, and *MGMT* promoter methylation may vary among different tissues. Based on these considerations it would be interesting to conduct a prospective trial studying *MGMT* expression in blood and tumour samples in order to understand the activity of temozolomide and its toxicity profile as this would be conducive to personalising drug delivery.

In June 2005, TMZ concomitant with radiotherapy followed by six cycles of maintenance chemotherapy became standard therapy worldwide (Stupp *et al*, 2005). The present study will therefore probably be the last classic phase II trial to be performed using an alternative TMZ schedule as first-line chemotherapy in chemonaïve and recurrent patients after standard radiotherapy. The findings made indicate that a phase III trial on a larger series should be launched in order to evaluate whether new chemotherapy regimens combining dose-intensity concepts with the manipulation of chemoresistance may yield a good risk/benefit/cost ratio in glioblastoma patients.

## Figures and Tables

**Table 1:** Characteristics of patients

	**No. of patients (%)**
*Gender*	
Female	13 (39)
Male	20 (61)
	
*Age* (*years)*	
Median	57
Range	31–71
	
*KPS*	
Median	90
Range	60–100
	
*Extent of resection*	
Gross total resection	13 (39)
Partial resection or biopsy	20 (61)
	
*Previous treatment*	
Primary resection	33 (100)
Radiotherapy	33 (100)
Chemotherapy	0
Repeat surgery for recurrence	1
	
*Cycles of temozolomide*	
⩽3	18
4–6	7
7–9	6
>10	2

**Table 2:** Toxicity per patient

	**Grade 1 no. of patients (%)**	**Grade 2 no. of patients (%)**	**Grade 3 no. of patients (%)**	**Grade 4 no. of patients (%)**
Neutropenia	1 (3)	2 (6)	2 (6)	2 (6)
Lymphopoenia	1 (3)	6 (18.2)	8 (24.2)	0
Thrombocytopenia	1 (3)	0	0	1 (3)
Anemia	5 (15.2)	1 (3)	1 (3)	0
Nausea	0	2 (6)	1 (3)	0
Constipation	4 (12.1)	4 (12.1)	1 (3)	0
Increased transaminase	5 (15.2)	1 (3)	1 (3)	0
Infection with lymphopoenia	1 (3)	3 (9.1)	1 (3)	0
